# Operation for Stricture, Followed by Uræmic Poisoning

**Published:** 1878-11

**Authors:** H. L. Wilson

**Affiliations:** Atlanta, Ga.


ATLANTA
### Medical and Surgical Journal.

Vol. XVI.] NOVEMBER—1878. [No. 8

### Original Communications.

### OPERATION FOR SRICTURE, FOLLOWED BY URÆMIC POISONING.

By H. L. WILSON, M.D., Atlanta, Ga.

September 7th, 1878, I operated on Alvin D., age 30, for strictures of the urethra. He had one at the meatus, another behind the corona glandis, and a third in the bulbous portion of the urethra. I could only introduce a No. 9 French bougie at first, but by gradual dilatation I increased the size sufficiently to introduce Civiale's urethrotome. After passing all of the strictures, I sprung the blade, and gently withdrew the instrument, cutting on a line with the frenum.

Immediately after the operation, I introduced, first, a No. 30, then 31, and at last 32 French bougie, with ease to the patient. I had purged his bowels the day before, and ordered x grs. quinine the night previous to the operation. I now gave quinine, grs. v, and ordered a warm hip bath, with positive instructions for him to remain in bed, which he did until September 12th. Each day I introduced the largest bougie, and left him all the time in splendid condition. On the 12th I allowed him to sit up in a rocker, on the 13th he came to my office, to have the instrument used, and announced himself well,—had no trouble whatever. I cautioned him to go slow, to do no work, and to be prudent in all things. On the 14th he failed to appear at my office, and failing also on the next day, I began to make inquiry, and found that he had been drinking;—about the same time I received a call to see Alvin, with the information that he was dying. Upon reaching his room, he informed me, that about two hours previous, he had attempted, while sitting in a chair, to introduce our instruments, being still under the influence of whisky: he succeeded only in bringing about excessive pain, inability to pass his urine, and considerable fever, —the first fever he had. His rigors were frequent and alarming. I gave him a hypodermic injection of morphine gr. $\frac{1}{2}$, and as hot a bath as could be borne; in about twenty minutes he relaxed and fainted; I immediately took him from the bath and placed him between blankets, with plenty of bed clothes on top to keep him comfortable. He said he felt good, and passed urine freely. On the morning of the 16th I found he had passed a comfortable night, voiding some urine about 4 o'clock in the morning. During the afternoon a cool stage came on, which lasted several hours, but did not amount to a distinct rigor. I gave him whisky and quinine every hour and a half to induce reaction, which eventually came in a very tardy and feeble way. He now had congestion of the kidneys, and by percussion over the region of the bladder I found there was no water present; for fifty-three hours he did not secrete one drop of water. Uræmia supervened, being a dry harsh skin, pulse 130 to 140, and delirium. I continued the whisky and quinine, and in addition gave bi carb. pot., grs. x, in Ponce de Leon water, every three hours; I pushed the water in interim.

At the expiration of time mentioned, he began to void very offensive decomposed urine, and in about twelve hours thereafter, reason gradually returned. At this juncture, there was some diaphoresis, with continuous fall of the pulse, until it reached the normal beat. I continued the treatment *pro re nata*, and in some ten or twelve days my patient was able to sit up, and improved each day to complete convalescence.

---

A synopsis of this case was reported to the Atlanta

Academy of Medicine, and published in the Oct. No. of the ATLANTA MEDICAL AND SURGICAL JOURNAL.

The case being one of great interest to me I have concluded to give more fully the particulars of it, and hence the article now presented.

